# Telocytes and Their Extracellular Vesicles—Evidence and Hypotheses

**DOI:** 10.3390/ijms17081322

**Published:** 2016-08-12

**Authors:** Dragos Cretoiu, Jiahong Xu, Junjie Xiao, Sanda M. Cretoiu

**Affiliations:** 1 Division of Cellular and Molecular Biology and Histology, Department of Morphological Sciences, Carol Davila University of Medicine and Pharmacy, Bucharest 050474, Romania; dragos@cretoiu.ro; 2 Victor Babeş National Institute of Pathology, Bucharest 050096, Romania; 3 Department of Cardiology, Tongji Hospital, Tongji University School of Medicine, Shanghai 200065, China; jhx_tj@hotmail.com; 4 Cardiac Regeneration and Ageing Lab, Experimental Center of Life Sciences, School of Life Science, Shanghai University, Shanghai 200444, China

**Keywords:** telocytes, telopodes, extracellular vesicles, exosomes, ectosomes

## Abstract

Entering the new millennium, nobody believed that there was the possibility of discovering a new cellular type. Nevertheless, telocytes (TCs) were described as a novel kind of interstitial cell. Ubiquitously distributed in the extracellular matrix of any tissue, TCs are regarded as cells with telopodes involved in intercellular communication by direct homo- and heterocellular junctions or by extracellular vesicle (EVs) release. Their discovery has aroused the interest of many research groups worldwide, and many researchers regard them as potentially regenerative cells. Given the experience of our laboratory, where these cells were first described, we review the evidence supporting the fact that TCs release EVs, and discuss alternative hypotheses about their future implications.

## 1. Introduction

**Figure 1:**
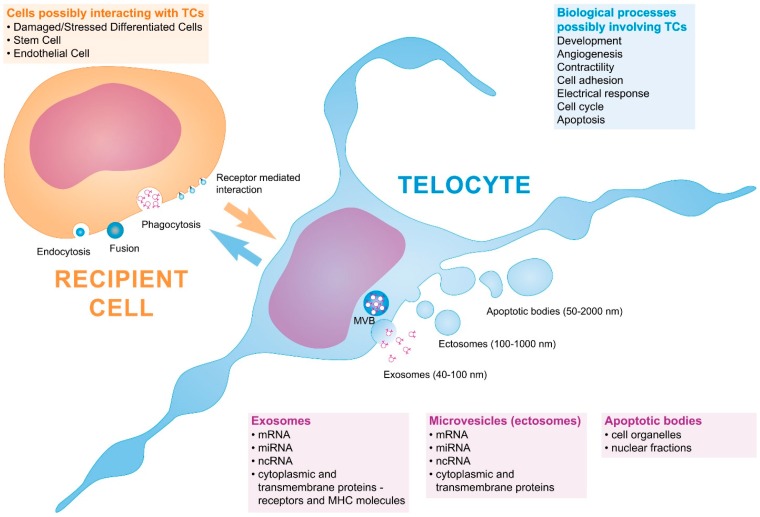
Schematic diagram of EVs transfer between cells, particularized for telocytes (TCs). Cells produce three types of extracellular vesicles (EVs): exosomes, ectosomes, and apoptotic bodies. The vesicles may be endocytosed, might fuse directly with the plasma membrane, or determine biological processes by ligand–receptor interactions on the cell surface. Arrows are indicative of the fact that the transfer is bidirectional and that EVs can shuttle between cells to communicate and exchange genetic material. Depending on the site of biogenesis, EVs’ heterogeneity, size, and composition are slightly different. ncRNA: non-coding RNA; miRNA: microRNA; MVB: multivesicular body.

Living cells communicate between themselves by different modalities, which are represented by cell junctions and the cell secretion of different soluble factors. The latter can act in an autocrine, paracrine, or endocrine manner. The last decade brought in a new evolutionary concept—that cellular communication can also be mediated by the transfer of genetic information [1]. This genetic transfer (e.g., mRNA, microRNA, long non-coding RNA, and occasionally genomic DNA) is intermediated by extracellular vesicles generated and released by prokaryotic and eukaryotic cells [2]. Extracellular vesicles (EVs) are nano-sized membrane-surrounded structures originating in the endosomal compartment or shed from the plasma membrane. Classified by their size and mechanisms of biogenesis, EVs can, in general, be categorized into three classes: (a) exosomes; (b) ectosomes or shedding microvesicles; and (c) apoptotic bodies. Exosomes were discovered almost three decades ago as “cell debris”, and have an endocytic origin and variable diameters between 30 and 100 nm [3,4]. Ectosomes (also known as microvesicles) have diameters between 100 and 1000 nm and form by direct budding from the plasma membrane [5]. Apoptotic bodies (50 nm–2 μm) are released by cells undergoing programmed cell death via outward blebbing of the apoptotic cell membrane. EVs carry receptors, bioactive lipids, proteins, and, most importantly, nucleic acids, such as mRNA, microRNA (miRNA), and non-coding RNAs. Their membrane composition (marker proteins) is particular according to the vesicle type, and their content is also variable [4,6] Telocytes are no exception to this mode of communication, being able to release and receive different types of vesicles (Figure 1).

The presence of EVs has been reported in interstitial spaces and in all biological fluids, including plasma, saliva, urine, cerebrospinal fluid, sputum, bronchial lavage fluid, malignant ascites, amniotic fluid, breast milk, and seminal fluid [7,8,9,10]. EVs were also detected as a heterogeneous population in the secretome from cells cultured in vitro, in conditioned media [11,12]. Protected by their external lipid bilayer, the content of EVs targets the recipient cells by three different mechanisms: direct fusion with their plasma membranes, receptor-mediated uptake, and endocytosis (phagocytosis) [13,14,15].

EVs have been found to have important roles in many important physiological processes, such as stem cell upkeep [16,17], tissue repair [18], immune surveillance [19] and vascular hemostasis [20]. Moreover, EVs seem to play an important role in several diseases, such as cancer, neurodegenerative, cardiovascular, and metabolic diseases [21,22,23,24,25,26].

Nowadays, the importance EVs in research is highlighted by the immense interest of the extracellular vesicle community, since EVs are considered as biomarkers, and also as drug, vaccine, and gene vector delivery tools in human diseases [27,28,29].

In this review, we summarize the recent research on the characterization of a new cell population within the stromal compartment, namely the telocytes (TCs). We also highlight the fact that TCs are able to release EVs, and we assess the research being carried out and the current progress examining the roles of these cells as communicating devices.

## 2. Telocytes as a Particular Type of Interstitial Cells

**Figure 2:**
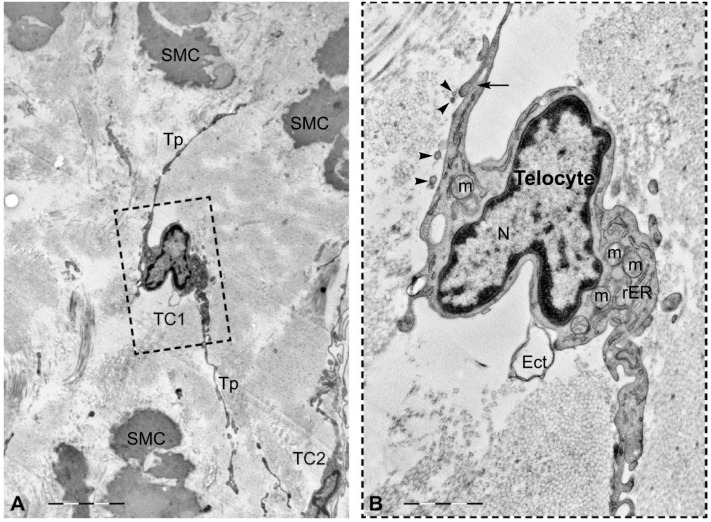
Transmission electron microscopy (TEM) of a telocyte in human non-pregnant myometrium. (**A**) Two cellular bodies (TC1, TC2) can be easily seen in the interstitial space between smooth myocytes. One telocyte has long, convoluting telopodes (TC2). Scale bar = 5 μm; (**B**) Higher magnification detail of the area marked with a dotted square in (**A**). Note that the heterochromatin is mostly confined to the periphery of the nucleus, but is also dispersed throughout. Scale bar = 1.5 μm. TC: telocyte; Tp: telopode; SMC: smooth muscle cell; m: mitochondrion; rER: rough endoplasmic reticulum; N: nucleus; arrowhead: exosome; Ect: ectosome; arrow: cellular junction.

**Figure 3:**
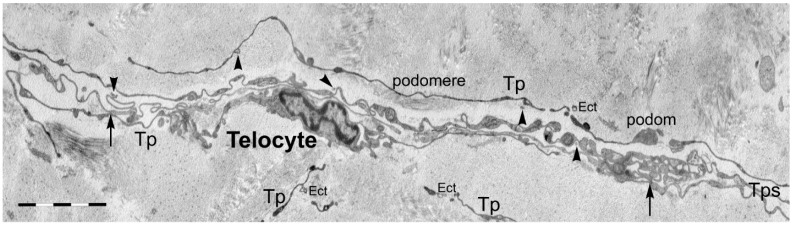
Transmission electron microscopy (TEM) of a telocyte in human non-pregnant myometrium. Image obtained by concatenation of seven microscopic fields. The telocyte exhibits a spindle-shape cell body, from where two extremely long telopodes are emerging. In the close proximity, other telopodes with tortuous trajectories contact the central telocyte by homo-cellular junctions, creating an intricate network. One can also observe numerous extracellular vesicles (arrowheads: exosomes; Ect: ectosomes) either shedding from or surrounding the telopodes. Arrows: cellular junctions; Tp(s) = telopode(s). Scale bar = 5 μm.

**Figure 4:**
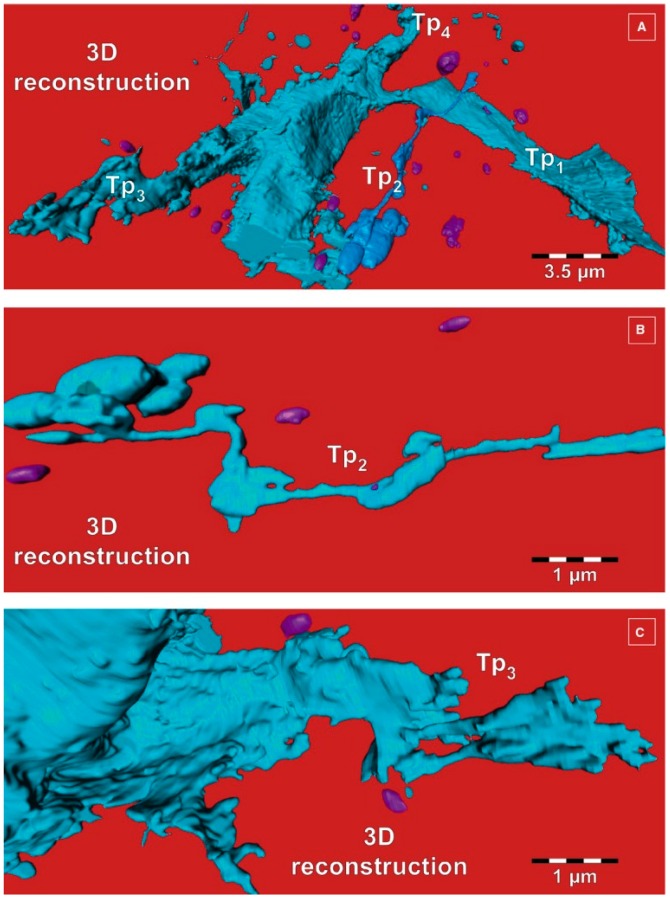
Focused ion beam scanning electron microscope (FIB-SEM) tomography. Three-dimensional reconstruction details of telopodes (Tps), from different viewing angles. (**A**) From this angle, four telopodes can be seen; (**B**) Tp2 has enlarged segments (podoms) alternating with slender segments; (**C**) Telopode with anfractuous contour. Extracellular vesicles appear in purple. Reproduced with permission from [35].

Telocytes (TCs) represent a recently-discovered cell population of the connective tissue (the stromal compartment forming the supportive framework of any organ) [30,31]. According to Popescu, their discoverer, the simplest description of TCs is cells with telopodes [32]. Telopodes are extremely long extensions (tens to hundreds of micrometers) which arise from the small cell body of TCs (Figure 2) [33]. Telopodes are characterized by a moniliform appearance in the bidimensional plane of ultrathin sections, with dilated portions called podoms and very thin regions named podomers (Figure 3). A three-dimensional perspective changes the first impression of telopodes, which appear to be long, flattened irregular veils and tubular structures with uneven caliber, because of irregular dilations corresponding to the podoms (Figure 4) [34,35].

Telocytes are nowadays seen as connecting devices, since numerous papers describe their ability to interact with themselves by homocellular junctions and with other cell types by heterocellular junctions (for details see review [36]). In addition, TCs also contact—directly or at a certain distance—important surrounding structures, such as blood vessels, nerve endings, smooth muscles, glandular elements, and covering epithelia [37,38].

Telocytes are functionally distinct from mesenchymal stem cells and fibroblasts with regard to their gene expression profile, and might have specific roles in cell signaling, tissue homeostasis, remodeling, and angiogenesis [39]. Chromosomal analysis also revealed that specific genes in lung TCs are different from those of pneumocytes, airway cells, mesenchymal stem cells, and lymphocytes [40,41,42,43,44]. Recently, TCs were characterized with the aid of various omics technologies such as mass spectrometry and multiplexed assays [45,46,47]. Several proteins were found to be up-regulated in TCs’ proteome—e.g., mitochondrial thioredoxin-dependent peroxide reductase, protein disulphide-isomerase A3, myosin-14, myosin-10, filamin-B, sodium/potassium-transporting ATPase subunit α-1 and keratin, type II cytoskeletal 1. These proteins are also regularly found in the proteome of mammalian extracellular vesicles, and therefore it has been proposed that TCs are involved in extracellular environment homeostasis, possibly influencing stem cell niches and leading to cell differentiation [47].

## 3. Telocytes and the Horizontal Transfer of Information

Telocytes—formerly known as interstitial Cajal-like cells (ICLC)—were shown to release EVs. Mandache et al. showed the presence of such vesicles soon after the first detailed ultrastructural characterization of ICLC [48]. Since that early study, which suggested the existence of a paracrine and/or juxtacrine intercellular mutual modulation between these special cells and the surrounding cells, much interest was dedicated to this type of intercellular communication. In the stromal space of different organs, other studies revealed the existence of EVs derived from the cellular body of TCs and also from their telopodes [38,49,50,51]. In addition, a morphometric comparison was performed between extracellular membranous vesicles (exosomes and shedding microvesicles) found in human non-pregnant and pregnant uterus [52]. In these two physiological conditions, exosome release seemed to be more pronounced in pregnancy, suggesting a horizontal transfer of important macromolecules among neighboring cells [52]. Telocytes have been shown to be implicated in a variety of human pathologies (as reviewed in [53]), where they are significantly reduced and altered. Therefore, we can consider that the release of EVs might also be affected in this context through altered intercellular signaling. Additionally, as based on their different immunohistochemical subtypes (suggesting organ-specific phenotypes of TCs [54,55]), their local and distal microcommunication mechanisms might also be diverse, including the content of the EVs.

**Figure 5:**
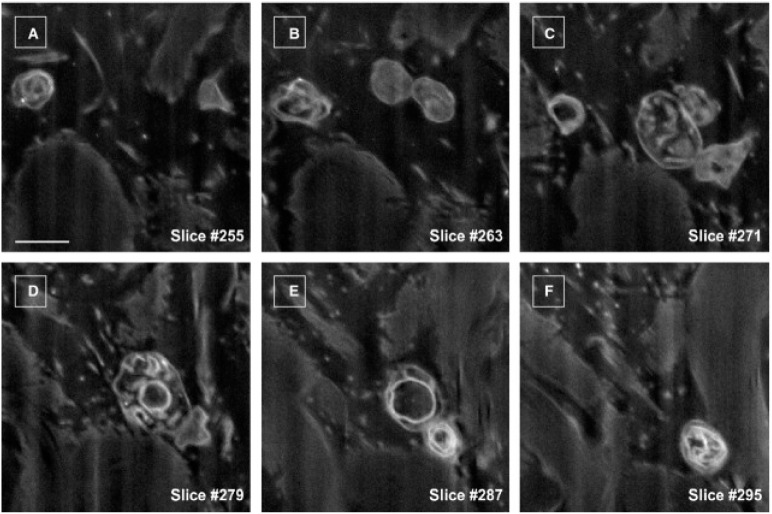
FIB-SEM of extracellular vesicle dynamics around a telocyte. (**A**–**F**) Six non-consecutive serial images depicting the biological fine structure of some EVs. Scale bar is 0.5 μm. Reproduced with permission from [35].

**Figure 6:**
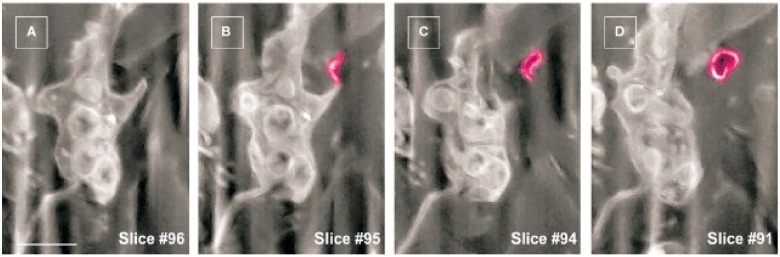
(**A**–**D**) FIB-SEM serial images of a human dermal telocyte presenting an extracellular vesicle (**purple**) budding from a podom. Note the empty appearance of the vesicle. Scale bar is 0.5 μm. Reproduced with permission from [35].

The release of EVs by TCs has also been demonstrated in vitro with the aid of electron microscopy and electron tomography. Fertig et al. described that cardiac TCs in culture release exosomes (45 ± 8 nm), ectosomes (128 ± 28 nm), and multivesicular cargos (MVC; 1 ± 0.4 μm) [56]. To gain insight into the third dimension of the arborescent conformation of TCs, focused ion beam scanning electron microscope (FIB-SEM) tomography was recently used to highlight human skin TCs. The 3D analysis of the reconstructed ultrastructural volume depicted the biological fine structure of some EVs (diameter 438.6 ± 149.1 nm, *n* = 30) at high resolution (Figure 5) [35]. The budding phenomenon was caught in progress, and represents valuable data about the three-dimensional morphology of telopodes and their capability to furnish extracellular vesicles at nanoscale dimensions (Figure 6).

It is known that the transfer of microRNA is mediated by EVs, which function as effective delivery vehicles. In fact, it has been shown that EVs are enriched in miRNAs and that secreted miRNAs are protected by the membrane structures of EVs [57,58]. Several miRNAs were reported as associated or not with TCs. In an effort to identify a biomarker for the identification of TCs, it has been demonstrated that the lack of miR-193 expression differentiates microdissected TCs from other stromal cells (3T3 fibroblasts) in cell culture. Moreover, they do not express any of the cardiomyocyte-specific miRNAs (miRs) (miR-1, 133a, or 208a). Instead, various levels of miR-21, 22, 29, and 199a-5p were detected, in accordance with TCs’ mesenchymal origin [59].

Recently, additional evidence has accumulated (in vivo and in vitro) about the importance of EVs in accomplishing the role of TCs in intercellular communication. Cismasiu and Popescu demonstrated the microRNA exchange between TCs and cardiac stem cells in cell cultures, when the EVs released from TCs are taken up by cardiac stem cells via endocytosis [60]. Furthermore, they demonstrated that cardiac stem cells deliver microRNA-loaded EVs to TCs, and suggested that “there is a continuous, post-transcriptional regulatory signal back and forth between TCs and stem cells” [60]. Several miRNAs with pro-angiopoietic potential (miR-126, miR-130, let-7e, and miR-100) were found to be expressed by TCs, and, moreover, the level of expression is increased in the myocardium soon after acute myocardial infarction [60]. Several experiments also reported an association between TCs and cardiac stem cells, stressing the contribution of TCs to neo-angiogenesis, especially in the infarcted myocardium [45,61,62]. This might also explain why cardiac TCs were found to be significantly increased in exercised heart, where they might contribute to cardiac renewal and regeneration [63]. The participation of TCs in angiogenesis is likely possible, since human lung telocytes could produce soluble factors such as VEGF and EGF, and were shown to induce the proliferation of pulmonary endothelial cells in cell cultures [64]. In a recent study, Li et al. [65] showed that both vascular TCs and vascular smooth muscle cells express miR-24, but the expression level of miR-24 is higher in TCs. Whether or not this miRNA is a cargo of the EVs and directly responsible for the effect remains to be established, but what is certain is that the supernatant of TCs in culture promoted the proliferation of vascular smooth muscle cells. Some other soluble factors in the supernatant—e.g., cytokines, including VEGF (vascular endothelial growth factor), IL-6 (interleukin-6), MIP-1α (macrophage inflammatory protein 1-α) might contribute to the repair process, too [45].

## 4. Future Directions

On one hand, there is a current growing interest in EVs is based on their physiological role in intercellular communication (especially in stem cell biology, where they can maintain the stemness capacity intervening in tissue repair) [66,67], and in pathological conditions (particularly in the pre-metastatic niche formation, cancer progression, and in the spread of numerous pathogens) [68,69,70]. On the other hand, the discovery of a new cell type known as telocytes, which are able to release EVs and interfere upon stem cells in their niches and upon other different somatic cells, allows us to speculate that they need special attention in the future. We need to learn more about the cargo in the EVs released by TCs, and if these vesicles are different in physiological and pathological conditions. Besides releasing vesicles, TCs were shown to have endocytic properties in the enteric wall (colon) and to participate in the uptake and storage of endogenous or exogenous particles in the skin and periodontal tissues (e.g., hemosiderin, melanin, and some components of dental amalgam). Therefore, Diaz-Flores et al. suggested that TCs are the principal non-macrophage cells with phagocytic-like properties [71]. As a consequence, one can consider that TCs represent important players in intercellular communication in between cells, locally or at a distance. A lot of information must be gathered before deciphering their precise role in physiological processes. In addition, TCs seem to change phenotype according to organ location [72,73,74,75], a phenomenon possibly explained by the different cargo in EVs and their shuttle trafficking between different cell types in response to diverse stimuli. Deciphering whether the content of EVs released/received by TCs is different according to location will open promising perspectives for controlling tissue homeostasis. It is possible that in the future we will be able to control the formation of telopodes [76], as well as the use of EVs as therapeutic potential agents.

As mentioned above, several hypotheses were raised about the functional roles of TCs; however, hardly any have been addressed to date. Although there are several papers discussing the interrelation between TCs and stem cells, the existing information about the involvement of TCs in cancer is scarce. Only two papers address this topic. Mirancea et al. [77] showed that TCs in normal dermis of the skin establish more heterocellular junctions in comparison with TCs of tumor dermis of basal cell carcinoma and squamous cell carcinoma, concluding that by decreasing their number of junctions, TCs might induce changes in intercellular communication into the peritumoral stroma and, consequently, into the whole tumor mass. Mou et al. stated that in situ “TCs communicate with breast cancer cells as well as other stromal cells, and might serve as a bridge that directly links the adjacent cells through membrane-to-membrane contact”, while in experimental conditions of a reconstituted breast cancer “TCs and other breast stromal cells facilitated the formation of typical nest structure, promoted the proliferation of breast cancer cells, and inhibited their apoptosis” [78].

In conclusion, TCs are cells capable of acting as integrators of many intercellular functions; however, there is a long way ahead until their functional capabilities are elucidated. Moreover, the specific cargo for their EVs must be characterized, and the biodistribution of these vesicles also remains to be established. TCs are seen by different groups as future targets with implications for regenerative medicine [79,80,81].
